# Airway bleed after percutaneous tracheostomy is not always procedure-related

**DOI:** 10.4103/0972-5229.56057

**Published:** 2009

**Authors:** Vijaya P. Patil, Amit Singhal, C. S. Pramesh

**Affiliations:** **From:** Departments of Anesthesiology and Thoracic Surgery, Tata Memorial Hospital, Parel, Mumbai 400 012, India

**Keywords:** Bleeding, complications, percutaneous tracheostomy

## Abstract

Tracheotomies are the most frequent surgical procedures performed in the intensive care unit. We present a case of major hemorrhage that occurred in the intensive care unit during an elective percutaneous dilational tracheostomy in a 46-year-old female diagnosed with multiple myeloma. The patient was later taken to the operation theatre and procedure-related cause of bleeding was ruled out. It was subsequently realized that the cause of bleeding was intrapulmonary and occurred coincidently with the tracheostomy.

## Introduction

Percutaneous tracheostomy is a reasonably safe procedure. In experienced hands, it is associated with a very low rate of complications comparable with open tracheostomy. Hemorrhage, though rare, should be attributed to the procedure especially if it occurs soon after the procedure. We describe a patient receiving chemotherapy for multiple myeloma who had significant bleeding following a percutaneous tracheostomy, which was unrelated to the procedure.

## Case Report

A 46-year-old female diagnosed with multiple myeloma was receiving thalidomide and dexamethasone. She presented to the hospital with breathlessness three days after the first cycle of chemotherapy.

**Figure 1:**
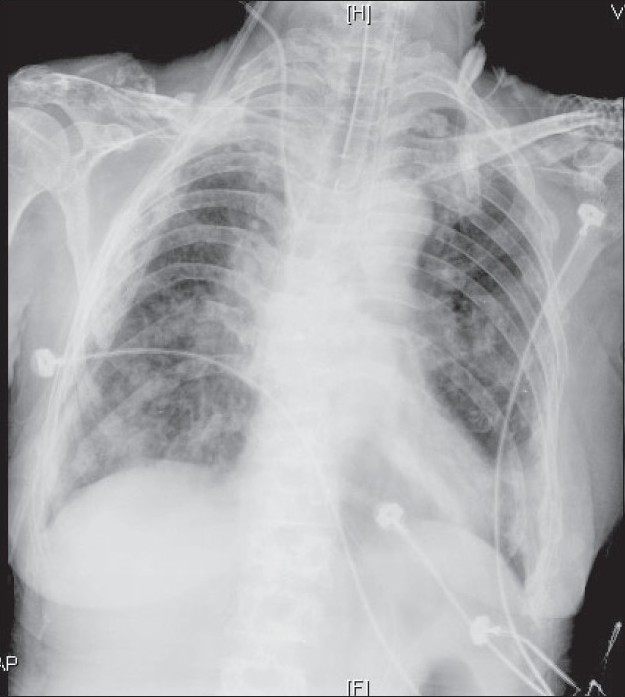
Chest radiograph immediately after intubation

In the ICU, she was afebrile with a respiratory rate of 22/min and bilateral coarse crepitations. She was saturating 88-90% on a high-concentration oxygen mask. Her chest X-ray [Figure 1] showed bilateral infiltrates. An echocardiography revealed normal cardiac function. She was diagnosed with community acquired pneumonia and started on antibiotics. She responded well to non invasive ventilation (NIV) initially but worsened after 10 hours requiring intubation and invasive ventilation. A tracheal aspirate culture confirmed a diagnosis of *Pneumocystis carinii* pneumonia (PCP) for which she was started on clindamycin and primaquine (renal dysfunction).

Three days later, she was successfully extubated. However, she worsened on the next day requiring reintubation, which was easy. A percutanous dilatational tracheostomy (PDT) was performed and a No. 7.0 cuffed tracheostomy tube was inserted. Before the procedure, her coagulation profile revealed normal APTT 22.3, INR 1.5, and platelet count 209 × 10^9^/L. She received 400 ml of fresh frozen plasma before the procedure. The procedure was uncomplicated with a smooth tracheostomy tube insertion and was done without a fibreoptic bronchoscope. However, soon after the tracheostomy, there was significant bleeding from the tracheostomy tube amounting to 75 ml, mixed with air. Suspecting a procedure-related bleeding, the tracheostomy tube was removed and an oral endotracheal tube was reinserted. The bleeding stopped soon after. The patient was taken to the operating room and the tracheostomy site was explored, which showed no evidence of bleeding or tracheal wall injury. The tracheostomy tube was reinserted. However, after 12 hours another episode of bleeding took place following which there was absent air entry in the left lung. The coagulation profile was repeated and showed platelets of 134 × 10^9^/L, APTT 25.1, and INR 1.23. Rigid bronchoscopy revealed that the trachea and left main bronchi were filled with large firm clots. There was no trauma to the trachea but the entire tracheobronchial tree looked inflamed with a slow continuous ooze from the left main bronchus. Some of the material removed looked firm and was therefore sent for a histopathological examination. A frozen section report was suggestive of plasmacytoma. The patient continued to have intermittent bleeding episodes along with passage of white fibrinous material. Over the next 4 days, her platelet count was in the range of 2.09 × 10^9^/L - 1.04 × 10^9^/L, INR between 1.3-1.57, APTT between 23.3-25.4, and WBC count between 3.6 × 10^9^/L-5 × 10^9^/L. In view of the disease involvement of the lung, the patent was restarted on high-dose corticosteroids and thalidomide. However, she progressively deteriorated and a decision was taken to limit her treatment in view of extensive disease.

## Discussion

Respiratory failure is a common problem in intensive care units (ICU) and a tracheostomy has become one of the most frequently performed procedures in critically ill patients. PDT has replaced the conventional tracheostomy in the majority of ICU patients as it is rapid and simple, performed at the bedside, and avoids complications associated with the transport of patients. In addition, the exclusion of surgical intervention improves the cost benefit ratio without compromising safety. With increasing familiarity with the procedure, indications for PDT have been extended to patients with previously defined contraindications, such as unfavourable anatomy, inability to extend the neck, and coagulopathy or use of anticoagulants.[1,2] In the immediate periprocedural period, hemorrhage is the most common complication rarely being fatal. Reported incidence of bleeding following PDT ranges from 0.5-5%.[3–6] In a majority of the cases, it is light bleeding and the most common causes are trauma to the anterior jugular vein and entry through the thyroid isthumus. This bleeding can be easily controlled by local pressure. Patients with previous neck or upper mediastinal surgery can pose problems due to distorted anatomy and adhesions where an ultrasound scan is useful. Shlugman, *et al*. have reported fatal arterial bleeding during the performance of PDT due to an avulsed right subclavian artery.[7] For bleeding arising from within the trachea, the most likely cause is a mucosal tear or a serious posterior tracheal wall injury, which warrants surgical exploration.

Our patient had a favorable neck anatomy, normal coagulation parameters, and a smooth procedure. Bleeding after PDT in our patient was in the form of blood mixed with air suggesting intrapulmonary origin rather than major airway or soft tissue. Neither surgical exploration of the tracheostomy site nor bronchoscpic examination showed any evidence of procedure-related damage ruling out trauma as the cause of bleeding.

The causes of pulmonary hemorrhage are numerous and autoimmune disorders form a large part of it due to capillary involvement. Autoimmune vasculitis, Goodpasture's syndrome, Wegener's granulomatosis, and SLE are some common causes. Though we did not perform an autoimmune screen, since the patient was taking immunosuppressants and high-dose corticosteroids, it is unlikely that the bleeding could have been because of the autoimmune process.

Leptospira is another cause of diffuse pulmonary hemorrhages and are generally seen during the monsoon season. We did not screen our patient for this possibility as our patient was afebrile throughout her stay with normal liver and renal functions.

There are case reports of extensive pulmonary bleeds secondary to Strongyloids hyperinfection in an immunocompromised patient.[8] This could have been a possibility in our patient though we did not investigate our patient for this.

Finally, the cause for alveolar hemorrhage in our patient could have been PCP. An association between PCP and diffuse pulmonary hemorrhage has been reported in 32% of HIV-positive patients of whom 13% had severe hemorrhage.[9] Until now, there has been only one case report of PCP and diffuse pulmonary hemorrhage in a non HIV-positive patient.[10] PCP may be one of possible causes of diffuse pulmonary hemorrhage.

In conclusion, one has to think of other possible causes of bleeding following PDT along with procedure-related bleeding as one of the main causes.
